# Metabolic syndrome, its components, and their association with dietary patterns among early adolescents in Sudan

**DOI:** 10.3389/fnut.2026.1939035

**Published:** 2026-09-09

**Authors:** Fatima A. Elfaki, Aziza I. G. Mukhayer, Mohamed E. Moukhyer, Rama M. Chandika, Husameldin E. Khalafalla, Stef P. J. Kremers

**Affiliations:** 1 Department of Clinical Nutrition, College of Nursing and Health Sciences, Jazan University, Jazan, Saudi Arabia; 2 Research Institute of Nutrition and Translational Research in Metabolism (NUTRIM), Faculty of Health, Medicine & Life Sciences, Maastricht University, Maastricht, Netherlands; 3 Department of Health Education and Promotion, Faculty of Health, Medicine & Life Sciences, Maastricht University, Maastricht, Netherlands; 4 School of Medicine, Ahfad University for Women, Omdurman, Sudan; 5 Public Health Programs, School of Medicine, University of Limerick, Limerick, Ireland

**Keywords:** adolescents, dietary pattern, factor analysis, metabolic syndrome, Sudan

## Abstract

**Background:**

The prevalence of global metabolic syndrome (MetS) is increasing alongside childhood obesity, and diet plays an important role in metabolic health.

**Objective:**

This study examined MetS, its components, and dietary pattern associations among Sudanese early adolescents aged 10–15 years (*N* = 921).

**Methods:**

In this cross-sectional study conducted in 2018–2019, dietary intake was assessed using a food frequency questionnaire; dietary patterns were derived by principal component analysis; and associations with MetS components and weight status were examined using the multiple linear regression analysis.

**Results:**

Three patterns emerged: starchy/animal-origin (starchy foods, fish, chicken, eggs, milk/dairy, meat), Western (fast/fried foods, snacks, soda drinks, sweets), and plant-based (legumes, vegetables, fruits, fresh juices) food patterns. Starchy/animal-origin and Western patterns were positively associated with weight status, and the plant-based pattern was inversely associated with weight status (all *p* < 0.01). After adjusting for gender, residence, age, and parental education, the starchy/animal-origin pattern was associated with greater waist circumference (WC; B = 2.05, 95% CI: 1.50, 2.60, *p* < 0.01) and higher systolic blood pressure (SBP; B = 0.62, 95% CI: 0.03, 1.21, *p* = 0.038). The Western pattern was associated with lower WC (B = −0.84, 95% CI: −1.52, −0.15, *p* = 0.016), whereas the plant-based pattern was associated with lower WC (B = −2.70, 95% CI: −3.28, −2.14, *p* < 0.01), lower SBP (B = −1.14, 95% CI: −1.75, −0.53, *p* < 0.01), and lower diastolic blood pressure (B = −0.68, 95% CI: −1.29, −0.07, *p* = 0.028).

**Conclusion:**

The starchy/animal-origin pattern showed adverse adiposity, while the plant-based pattern showed more favorable profiles among Sudanese early adolescents. School-based dietary interventions are needed to promote balanced eating and reduce MetS risk.

## Introduction

Sudan, situated in sub-Saharan Africa, is home to a diverse array of tribes and sub-tribes across rural and urban landscapes. As a result, there is a wide range of food consumption habits and nutritional practices across Sudan’s several states. Considerable differences in climatic conditions, agricultural production, food accessibility and availability, and demographic preferences are represented in the complexity of Sudan’s culinary landscape. Additionally, wealth plays a significant role in determining dietary diversity levels (1). Diet, a vital component of lifestyle, has been consistently associated with metabolic syndrome (MetS) (2–5). Dietary patterns, defined as the proportions, quantities, and combinations of a variety of foods, drinks, and nutrients in diets, along with the habitual frequency of their consumption, play a crucial role in shaping health outcomes (6). In Sudan, adolescents’ diets are undergoing a nutrition transition, with a growing reliance on refined starchy staples, fast and fried foods, snacks, and sugary drinks alongside traditional foods, while intake of fruits, vegetables, and legumes remains comparatively low; affordability and market access are key determinants of these intake patterns (7). This shifting dietary landscape has coincided with a rising burden of MetS-related morbidity among Sudanese early adolescents: In Khartoum State, MetS has been reported to affect 2.3% of early adolescents, with a considerably higher prevalence of individual components such as elevated waist circumference and dyslipidemia (8).

Numerous studies have explored unhealthy dietary patterns among students (9–13), including studies that specifically examine the association between dietary patterns and MetS in adolescents. It has been reported that more westernized dietary habits (i.e., those characterized with a high intake of fast foods and added sugars) can increase the risk of MetS (14), whereas diets rich in fruits and vegetables might have a protective effect (15). MetS is rapidly increasing alongside rising childhood and adolescent obesity and the increasing prevalence of unhealthy dietary patterns among youth (8, 16, 17). Recognizing the role of a healthy diet in children and adolescents, studies indicate that it not only reduces the risk of chronic diseases but also promotes healthy growth and development (18). Dietary habits may influence hormonal changes during the pubertal stage of adolescence (7). Consequently, identifying dietary patterns may be crucial for characterizing unhealthy food consumption and developing strategies to improve eating habits, thereby reducing the risk of chronic diseases (19). Dietary pattern analysis has been identified as a valuable approach to assessing the relationship between overall diet quality and health outcomes (20). It may provide an optimal explanation of the relationship between dietary pattern and health outcomes by focusing on the entirety of a diet rather than isolating the health effects of a single food or nutrient (21). Studies exploring dietary patterns and their relationships with MetS have been advocated for children and adolescents, but such studies have been conducted infrequently, and further research is still needed (22).

Considering the importance of diet in health, particularly during early adolescence, and acknowledging the limited existing data on MetS and its components’ association with dietary patterns in Sudan, the present study aims to assess the association between MetS and its components and dietary patterns among Sudanese early adolescents aged 10–15 years old.

## Materials and methods

### Study design and population

A cross-sectional study was conducted from January 2018 to December 2019 in Khartoum State, Sudan, among school-going early adolescents aged 10–15 years. Khartoum State was selected as the study site because it is the most populous and urbanized state in Sudan, encompassing both urban and rural localities and thereby enabling socioeconomic and geographical diversity in the sample. A two-stage, stratified cluster systematic random sampling method was used to obtain a representative sample of school-going adolescents across contrasting residential environments. In the first stage, localities within Khartoum State were stratified by residence type (urban vs. rural). Khartoum locality was selected to represent urban settings, and Karary locality was selected to represent rural settings by the ballot method. Within each selected locality, schools were enumerated and used as primary sampling units (clusters). A total of 21 schools were selected using simple random sampling proportional to the target sample size: 12 from Khartoum locality (urban) and 9 from Karary locality (rural). In the second stage, complete student rosters were obtained from each selected school, and participants were systematically sampled by selecting every third student on the list. Eligibility criteria were as follows: age of 10–15 years, enrolment in a selected school, and provision of written informed parental consent and student assent. Students without parental consent, those absent on all scheduled data collection days, or those with incomplete data on key variables were excluded. When a selected student was ineligible or declined to participate, the next eligible student on the roster from the same school was recruited to maintain the sampling interval and preserve the representativeness of the sample. A total of 1,064 students were initially approached; 921 adolescents (388 boys and 533 girls) were included in the final analysis after excluding those with incomplete data, yielding a response rate of 86.6%.

### Data collection

This study included a questionnaire to collect sociodemographic and food-frequency information from students through interviews conducted by trained data collectors. A pilot study was conducted among 50 students to assess the clarity and comprehension of the questionnaire items. The final version of the questionnaire was prepared in both Arabic and English. Additionally, well-trained technicians performed anthropometric and blood pressure measurements. Furthermore, laboratory technicians collected fasting blood samples to determine the concentrations of high-density lipoprotein cholesterol, triglycerides, and glucose.

### Sample size

An earlier study from Sudan reported that the prevalence of MetS was 2.3% (8) among early adolescents. At a 5% level of significance and a 1% margin of error, with a design effect of one, the required minimum sample size was 863. Furthermore, the minimum number of schools (clusters) was calculated as 21, with the assumption that the minimum size of schools was 45. However, to increase precision and account for non-response, 921 early adolescents were recruited into the study.

### Metabolic syndrome definition

The International Diabetes Federation (IDF) guidelines (23) were used to define metabolic syndrome. According to these criteria, children are classified as having metabolic syndrome if their waist circumference (WC) is ≥ 90th percentile and they exhibit two or more of the following criteria: triglycerides (TG) ≥ 150 mg/dL; high-density lipoprotein cholesterol (HDL-C) < 40 mg/dL; systolic blood pressure (SBP) ≥ 130 mmHg or diastolic blood pressure (DBP) ≥ 85 mmHg; and fasting plasma glucose (FBG) ≥ 100 mg/dL (23).

### Anthropometric measurement

Weight was measured with participants wearing light clothing and no shoes using a digital scale (SECA) with an accuracy of 0.1 kg. A portable stadiometer with a precision of 0.5 cm was used to measure height while participants stood barefoot. Measured height and weight were used to determine body mass index (BMI) (weight [kg]/height [m^2^]). Overweight and obesity were defined using BMI-for-age and height-for-age (z-score) based on age- and sex-specific BMI cutoff points developed by the World Health Organization (24). Waist circumference was measured in centimeters (cm) using an unstretched measuring tape to approximately 0.1 cm. Age-wise percentiles for both genders for WC were calculated separately, and early adolescents were categorized as normal or high WC using age-wise WC 90th-percentile cutoffs for both boys and girls (25).

### Blood pressure measurement

An automatic digital blood pressure monitor was used to measure systolic and diastolic blood pressure while participants were seated. Each measurement was repeated twice with a 5-min interval, and the average was recorded. Blood pressure was classified as elevated when SBP was ≥130 mmHg or DBP was ≥85 mmHg (23).

### Laboratory data

All participants were required to fast for at least 8 h the night before blood collection, and fasting status was confirmed by data collectors through a structured question at the time of blood draw. Blood samples were allowed to clot at room temperature before being centrifuged (L500 Tabletop Low Speed Centrifuge) for 10 min at 3,000 rpm to separate the serum, which was then transferred to the laboratory. Quality control measures were implemented to ensure the accuracy of the analytical tests. Serum levels of total TG, HDL-C, and FBG (mg/dL) were measured using an A25 analyzer (Biosystems SA, Costa Brava 30, Barcelona, Spain) and appropriate conventional laboratory reagents and enzymatic and calorimetric techniques. Standard enzymatic kit methods using a fully automated analyzer were used to assess TG, HDL-C, and FBG.

### Dietary assessment

Dietary information was collected through the food frequency questionnaire (FFQ). To minimize recall bias, the recall period was restricted to 1 week. Participants selected from six structured frequency options: never, once a week, 2–4 times a week, 5–6 times a week, 7–8 times a week, and 9 or more times a week. The food frequency items had been adopted with minor changes from an earlier study in Sudan (7).

### Dietary patterns analysis

Dietary patterns were derived using principal component analysis (PCA) applied to 14 predefined food groups, constructed by aggregating individual food items based on nutritional similarity and customer consumption patterns. The suitability of the data for PCA and the measure of sampling adequacy was confirmed by the obtained Kaiser–Meyer–Olkin (KMO) value of 0.834 and Bartlett’s test of sphericity (*p* < 0.01), both of which supported factorability of the correlation matrix (26). Components were retained based on a combination of eigenvalues >1.0, visual inspection of the scree plot, and interpretability of the factor structure. To enhance interpretability, an orthogonal varimax rotation was applied (27, 28). Food groups with absolute factor loadings ≥0.40 (29) were considered to contribute meaningfully to a given dietary pattern. In cases of cross-loading, food groups were assigned to the component on which they had the highest loading, provided the difference between loadings was sufficient; otherwise, they were excluded from the pattern interpretation. Each retained component was labeled according to the food groups with the highest loadings. Factor scores were calculated for each participant for all identified dietary patterns, with higher scores indicating greater predominance of the corresponding pattern.

### Statistical analysis

IBM Statistical Package for Social Sciences (SPSS) version 27 and R software (Version 4.5.3) were used for analysis and graph construction, respectively. Categorical variables were reported descriptively as percentages, and chi-square tests were used to assess significance. Factor analysis was conducted, and graphs were constructed with the psych and ggplot2 R packages, respectively. Multiple linear regression models were adjusted for confounders: age, gender, residence, and parental education level. A two-tailed *p*-value of < 0.05 was considered to be statistically significant.

### Ethical considerations

The study received approval from the Research Ethics Committee of the Federal Ministry of Health, Sudan (Ref. No. 0001 date 01-12-2017). Written informed consent was obtained from both parent and students to participate in the study.

## Results

### General background and metabolic characteristics of the study participants

Table 1 shows the participants’ general and metabolic characteristics. Participants were categorized by the diversity of their living areas. A notable majority (63.7%) of boys lived in urban areas, while more than half of the girls (53.5%) lived in rural areas. The difference in living areas between genders was statistically significant (*p* < 0.01). Regarding the parental educational level, over two-thirds of urban early adolescents had parents with university/postgraduate education, whereas more than half of rural early adolescents had illiterate parents. The difference in parents’ education levels between living areas was highly statistically significant (*p* < 0.01). Concerning weight status, underweight early adolescents were significantly more prevalent in rural areas, while overweight or obese early adolescents were significantly more prevalent in urban areas (*p* < 0.05). Among the participants, the prevalence of MetS was 2.3%.

**Table 1:** Background and metabolic characteristics by residence among Sudanese early adolescents.

Variables	*N* (%) (*n* = 921)	Urban *n* = 495 (53.7%)	Rural *n* = 426 (46.3%)	*^¥^p*-value
Gender				
Boys	388 (100.0%)	247 (63.7%)	141 (36.3%)	0.001^**^
Girls	533 (100.0%)	248 (46.5%)	285 (53.5%)	
Father’s educational level				
Illiterate	85	36 (42.4%)	49 (57.6%)	0.001^**^
Primary	160	64 (40.0%)	96 (60.0%)	
Intermediate	52	26 (50.0%)	26 (50.0%)	
Secondary	217	113 (52.1%)	104 (47.9%)	
University/postgraduate	302	206 (68.2%)	96 (31.8%)	
Do not know	105	50 (47.6%)	55 (52.4%)	
Mother’s educational level				
Illiterate	60	23 (38.3%)	37 (61.7%)	0.001^**^
Primary	102	40 (39.2%)	62 (60.8%)	
Intermediate	44	16 (36.4%)	28 (63.6%)	
Secondary	161	87 (54.0%)	74 (46.0%)	
University/postgraduate	380	248 (65.3%)	132 (34.7%)	
Do not know	174	81 (46.6%)	93 (53.4%)	
Weight status				
Underweight	153 (16.6%)	73 (47.7%)	80 (52.3%)	0.018^*^
Normal	573 (62.2%)	299 (52.2%)	274 (47.8%)	
Overweight	108 (11.7%)	66 (61.1%)	42 (38.9%)	
Obese	87 (9.4%)	57 (65.5%)	30 (34.5%)	
MetS and components				
MetS: yes	21 (2.3%)	17 (81.0%)	4 (19.0%)	0.011^*^
MetS: no	900 (97.7%)	478 (53.1%)	422 (46.9%)	
Normal FBG	314 (34.1%)	153 (48.7%)	161 (51.3%)	0.025^*^
High FBG	607 (65.9%)	342 (56.3%)	265 (43.7%)	
Normal TG	857 (93.1%)	455 (53.1%)	402 (46.9%)	0.143
High TG	64 (6.9%)	40 (62.5%)	24 (37.5%)	
Normal HDL-C	783 (85.0%)	406 (51.9%)	377 (48.1%)	0.006^*^
Low HDL-C	138 (15.0%)	89 (64.5%)	49 (35.5%)	
Normal BP	878 (95.3%)	477 (54.3%)	401 (45.7%)	0.111
High BP	43 (4.7%)	18 (41.9%)	25 (58.1%)	
Normal WC	839 (91.1%)	439 (52.3%)	400 (47.7%)	0.006*
High WC	82 (8.9%)	56 (68.3%)	26 (31.7%)	

MetS: metabolic syndrome, FBG: fasting blood glucose, TG: triglycerides, HDL-C: high-density lipoprotein cholesterol.

BP: blood pressure, WC: waist circumference, ¥: chi-square test, *significant and **highly significant.

### Reliability and optimal number of dietary patterns

**Figure 1:**
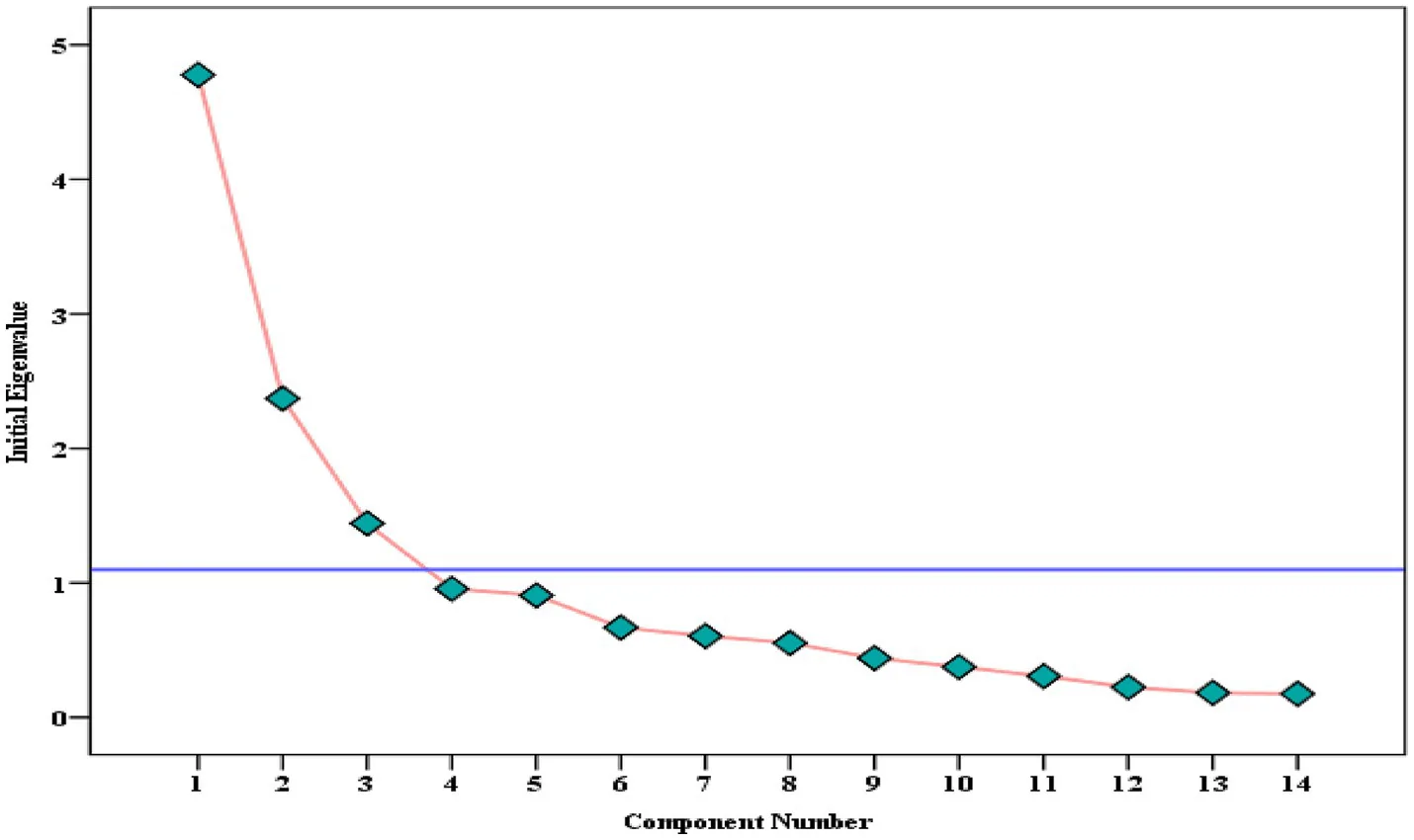
Scree plot.

As shown in the scree plot (Figure 1), three dietary patterns were above the absolute line, with an initial eigenvalue being 1.1.

### Factor loadings and communalities

Three dietary patterns were identified by the principal component analysis of the reported consumption of 14 food groups among Sudanese early adolescents, which together accounted for 61.38% of the total variance in the dataset. Three patterns could explain 25.51%, 23.27%, and 12.60% of the variance, respectively. The first dietary pattern was named the starchy/animal-origin food patterns and was characterized by high factor loadings for starchy foods, fish, chicken, eggs, milk/milk products, and meat. The second dietary pattern was named the Western food patterns, characterized by high factor loadings of fast and fried foods, snacks, soda drinks, and sweets. Similarly, the third dietary pattern was named plant-based food patterns, characterized by high factor loadings of legumes, vegetables, fruits, and fresh fruit juices (Table 2).

**Table 2:** Loadings for the three extracted dietary patterns.

Food items	Dietary patterns			Communalities
	Starchy/animal-origin foods	Western foods	Plant-based foods	
Starchy foods	0.87			0.77
Fish	0.87			0.76
Chicken	0.83			0.70
Eggs	0.77			0.62
Milk/milk products	0.60			0.46
Meat	0.52			0.42
Fast and fried foods		0.91		0.84
Snacks		0.86		0.76
Soda drinks		0.85		0.75
Sweets		0.82		0.70
Legumes			0.76	0.58
Vegetables			0.75	0.562
Fruits			0.48	0.35
Fresh fruit juices			0.40	0.32
% of variance	25.51	23.27	12.60	—
% of cumulative variance	25.51	48.78	61.38	—

Factor analysis: principal component analysis with the Kaiser criterion and varimax rotation was applied.

### Dietary patterns and body mass index

**Figure 2:**
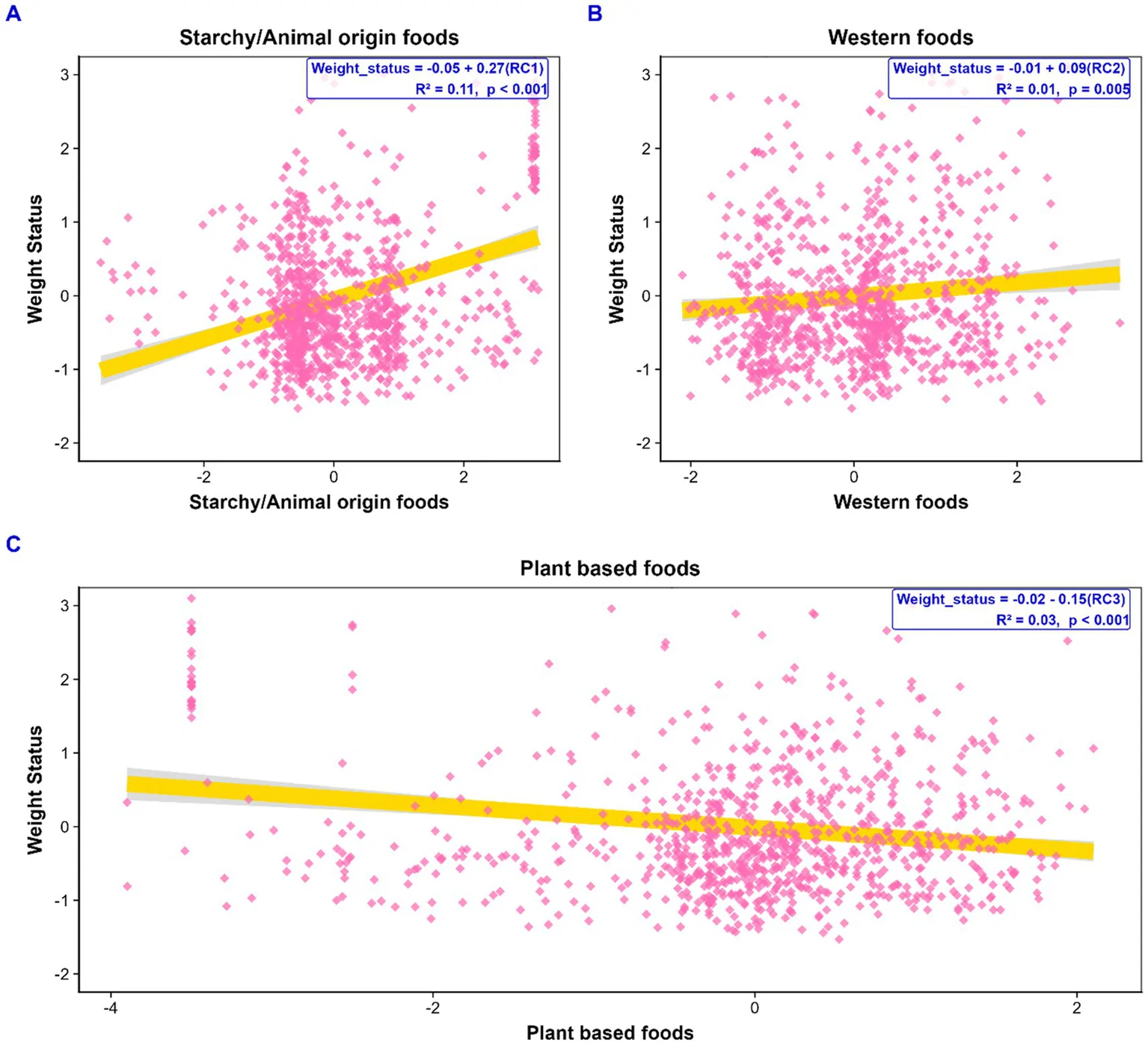
Associations between dietary patterns and weight status. **(A)** Starchy/Animal origin food. **(B)** Western foods. **(C)** Plant based foods.

Figure 2 presents the relationships between the three dietary patterns and weight status among Sudanese school-going early adolescents based on a multiple linear regression analysis. A highly significant positive association was observed between starchy/animal-origin food patterns and weight status (*β* = 0.27, *p* < 0.001, *R*^2^ = 0.11). This indicates that higher consumption of foods characterized by starchy/animal-origin food patterns is associated with increased weight status. The Western food patterns also showed a highly significant positive association with weight status (*β* = 0.09, *p* = 0.005, *R*^2^ = 0.01). This suggests that higher consumption of Western foods is associated with increased weight status. In contrast, the plant-based food patterns demonstrated a highly significant inverse association with weight status (*β* = −0.15, *p* < 0.001, *R*^2^ = 0.03). This indicates that high consumption of plant-based foods is associated with lower weight status.

Multiple linear regression model; starchy/animal-origin food patterns (RC1), Western food patterns (RC2), plant-based food patterns (RC3). Confounders (gender, age, residence, and parental education level) were adjusted.

### Dietary patterns and their associations with general characteristics

Table 3 shows the general characteristics and their associations with dietary patterns. A multiple regression analysis showed that boys had significantly higher consumption of starchy/animal-origin foods (*p* < 0.01), whereas girls had significantly higher consumption of plant-based foods (*p* < 0.01). No significant gender difference was observed in Western foods consumption (*p* = 0.875). Urban early adolescents consumed significantly more starchy/animal-origin foods (*p* < 0.05) than rural adolescents. Fathers’ education level was not associated with dietary patterns. Mothers’ education showed significant associations with both plant-based foods and starchy/animal-origin foods consumption: children of university/postgraduate-educated mothers had higher consumption of plant-based foods (*p* < 0.01) and, to a lesser extent, of starchy/animal-origin foods (*p* < 0.05), whereas no significant association was observed between mothers’ education and western foods consumption (*p* = 0.149) (Table 3).

**Table 3:** Association between dietary patterns and general characteristics among early adolescents.

Variables	Starchy/animal-origin foods				Western foods				Plant-based foods			
	B	Std. error	Beta	*p*-value	B	Std. error	Beta	*p*-value	B	Std. error	Beta	*p*-value
Gender	−0.201	0.067	−0.099	0.003	0.011	0.068	0.005	0.875	0.188	0.068	0.093	0.005
Residence	−0.165	0.068	−0.083	0.015	−0.121	0.069	−0.060	0.080	−0.114	0.068	−0.057	0.097
Age	0.019	0.075	0.009	0.796	−0.049	0.076	−0.022	0.516	0.025	0.075	0.011	0.741
Father’s education level	−0.025	0.024	−0.036	0.316	−0.021	0.025	−0.030	0.407	−0.010	0.025	−0.015	0.683
Mother’s education level	0.051	0.023	0.079	0.029	0.034	0.024	0.052	0.149	0.069	0.024	0.106	0.004

Multiple linear regression. B-slope coefficient, Beta-standardized B, std. error-standard error, *p* < 0.01 highly significant, *p* < 0.05 significant. Gender (boys = 1, girls = 2), residence (urban = 1, rural = 2), age (scale variable), father and mother educational level, (illiterate = 1, Khalwa = 2, primary = 3, intermediate = 4, secondary = 5, university = 6, and do not know = 7).

### Dietary patterns and associations with MetS components

Table 4 presents the associations between dietary patterns and metabolic syndrome components among Sudanese early adolescents (*n* = 921). Starchy/animal-origin food patterns demonstrated highly significant positive associations with waist circumference (B = 2.05; 95% CI: 1.50, 2.60; *p* < 0.01) and SBP (B = 0.62; 95% CI: 0.03, 1.21; *p* < 0.05), indicating that higher consumption of starchy/animal-origin food patterns was associated with increased central adiposity and elevated blood pressure. Western dietary pattern was significantly associated with lower WC (B = −0.84; 95% CI: −1.52, −0.15; *p* < 0.05), with modest negative trends observed for diastolic blood pressure (DBP) (B = −0.21) and fasting blood glucose (B = −0.13), though these did not reach significance. In contrast, the plant-based food patterns showed highly significant inverse associations with WC (B = −2.70; 95% CI: −3.28, −2.14; *p* < 0.001), SBP (B = −1.14; 95% CI: −1.75, −0.53; *p* < 0.001) and DBP (B = −0.68; 95% CI: −1.29, −0.07; *p* < 0.05); however, it was significantly and positively associated with FBG (B = 0.69; 95% CI: 0.04, 1.33; *p* < 0.05), an unexpected finding that runs counter to the otherwise protective pattern and warrants further investigation. None of the three dietary patterns showed a statistically significant association with triglycerides (starchy/animal-origin foods: *p* = 0.118; Western foods: *p* = 0.597; plant-based foods: *p* = 0.496) or with HDL-C (starchy/animal-origin foods: *p* = 0.791; Western foods: *p* = 0.229; plant-based foods: *p* = 0.748).

**Table 4:** Association of metabolic syndrome components with dietary patterns among Sudanese early adolescents (*n* = 921).

Variable	Starchy/animal-origin foods	Western	Plant-based
	Regression coefficient (B) (95% CI) *p*-value		
WC	2.05 (1.50, 2.60) 0.000^**^	−0.84 (−1.52, −0.15) 0.016^*^	−2.70 (−3.28, −2.14) 0.000^**^
SBP	0.62 (0.03, 1.21) 0.038^*^	−0.44 (−1.15, 0.27) 0.226	−1.14 (−1.75, −0.53) 0.000^**^
DBP	−0.18 (−0.77, 0.40) 0.538	−0.21 (−0.92, 0.49) 0.550	−0.68 (−1.29, −0.07) 0.028^*^
FBG	0.04 (−0.58, 0.66) 0.904	−0.13 (−0.87, 0.61) 0.730	0.69 (0.04, 1.33) 0.037^*^
TG	−1.41 (−3.18, 0.36) 0.118	0.58 (−1.56, 2.71) 0.597	−0.64 (−2.49, 1.21) 0.496
HDL-C	0.09 (−0.58, 0.76) 0.791	−0.50 (−1.31, 0.31) 0.229	−0.12 (−0.82, 0.59) 0.748

WC: waist circumference, SBP: systolic blood pressure, DBP: diastolic blood pressure, FBG: fasting blood glucose, TG: triglycerides, HDL-C: high-density lipoprotein cholesterol, CI: confidence interval, *Significant and **highly significant. Confounders: gender (boys = 1, girls = 2), residence (urban = 1, rural = 2), age (scale variable), father’s and mother’s education level (illiterate = 1, Khalwa = 2, primary = 3, intermediate = 4, secondary = 5, university = 6, and do not know = 7) adjusted using the multiple regression analysis.

## Discussion

The present study addressed the association between metabolic syndrome (MetS) and its components and explored dietary patterns among Sudanese early adolescents aged 10–15 years. Among Sudanese early adolescents, three dietary patterns were identified, including starchy/animal-origin foods, Western foods, and plant-based foods. Various cohort studies reported similar dietary patterns with the labels unhealthy (high-fat), western patterns, and healthy/prudent patterns (30–32). Earlier studies identified a number of dietary patterns ranging from 3 to 7 in factor analytical approaches (33–37).

Among the three dietary patterns, the starchy/animal-origin food patterns were common among urban early adolescents, especially among boys. This urban predominance may be attributed to greater household purchasing power and easier market access to animal-source foods and starchy convenience items in Khartoum locality, whereas households in the rural Karary locality more often rely on lower-cost, home-produced staples, reflecting the influence of income and food accessibility on dietary diversity in Sudan (1). Plant-based food patterns were notably high among girls, especially older ones. This age gradient may reflect greater health and body-image consciousness as girls approach mid-adolescence, together with increasing involvement in household food selection and meal preparation as they grow older. These results align with previous studies indicating that women are more likely to adopt a healthy dietary pattern (31, 37, 38). A plant-based food pattern was more prevalent among children whose mothers had a university/postgraduate education level. Moreover, the fruits and vegetables pattern showed an association with educational level; previous studies have also identified a higher educational level to be associated with a higher likelihood for a person to follow this pattern and make healthier food choices (31).

In the present study, early adolescents with higher consumption of starchy/animal-origin and Western foods were associated with increased weight status. Plant-based food patterns exhibited a negative association with weight status. These findings align with existing literature that high-protein and Western food patterns are associated with an increased risk of overweight and obesity (34, 39–43).

The first dietary pattern, named starchy/animal-origin foods in our study, which is rich in starchy foods, fish, chicken, eggs, milk/milk products, and meat, showed a significant positive association with two MetS components, waist circumference and systolic blood pressure; associations with diastolic blood pressure, fasting blood glucose, triglycerides, and HDL-C were not significant. This dietary pattern contains high amounts of animal meats (including processed meat and red meat) and high amounts of fat (44), especially saturated fats (45). The second dietary pattern, named the western food patterns in our study, was characterized by high factor loadings of fast and fried foods, snacks, soda drinks, and sweets. Contrary to our expectations, it was significantly and inversely associated with waist circumference and showed non-significant negative associations with the remaining MetS components. Consumption of Western foods, which have high energy density and low nutritional value, is generally believed to lead to excessive energy accumulation, obesity, and other metabolic diseases (46). Regular consumption of fast foods and sweet snacks could contribute to weight gain and insulin resistance (47, 48), playing a key role in MetS development in general populations; however, this pattern was not associated with an adverse MetS risk profile in the present sample. The third dietary pattern, termed plant-based food patterns, is characterized by high factor loadings of legumes, vegetables, fruits, and fresh fruit juices. It demonstrated a significant negative (protective) association with waist circumference, systolic blood pressure, and diastolic blood pressure; however, it showed an unexpectedly significant positive association with fasting blood glucose, while its associations with triglycerides and HDL-C were not significant. This pattern is recognized as one of the most common dietary components, boasting numerous health benefits. Consistent with previous studies, the “prudent” pattern showed a negative association with waist circumference (49–51).

## Limitations

The main limitation of this study lies in the cross-sectional nature of the data, which prevents us from drawing causal conclusions. The study area, limited to two localities, may lead to selection bias. In addition, the food consumption data collected through the food frequency questionnaire may not be representative of the entire year’s consumption. The lack of information regarding physical activity could affect both outcomes: MetS components and dietary patterns. The food frequency questionnaire relied on self-report and had not been formally validated against a reference method such as weighed food records or biomarkers in this population, which may have introduced recall and social desirability biases despite the short 1-week recall period. The high prevalence of elevated fasting blood glucose observed (65.9%), defined using the IDF cut-off of FBG ≥ 100 mg/dL, was higher than that reported in comparable adolescent populations; although fasting status was confirmed at the time of blood draw, incomplete adherence to the overnight fasting instruction cannot be entirely excluded and may have contributed to this finding, which should be interpreted with this caveat in mind. Additionally, data collection took place between January 2018 and December 2019; these findings therefore reflect the nutritional and socioeconomic situation in Khartoum State prior to the armed conflict that began in April 2023, which has since caused substantial disruption to schooling, food security, and healthcare access in the region. School-based intervention recommendations arising from this study should be interpreted in light of this changed context and may need to be re-evaluated once the current situation stabilizes.

Well-designed, large-scale prospective studies are warranted to overcome these limitations. Despite these limitations, our study had several strengths. Primarily, utilizing scientific sampling methods and rigorous quality control measures, and using anthropometric and physical assessments by trained assistants in a large sample of an understudied target population are particular strengths of this study. These features increase the study’s internal validity. However, as the sample was drawn only from school-going early adolescents in Khartoum State, the findings may not be broadly generalizable beyond this group, and caution is warranted when extending them to non-school-going adolescents or to other regions of Sudan. Furthermore, we controlled for potential confounders in our analysis as much as possible, including gender, age, residence, and parents’ education level, which play important roles in disease development.

## Conclusion

This study identified three distinct dietary patterns among Sudanese early adolescents. The starchy/animal-origin food patterns were predominant, followed by the western foods and plant-based food patterns. A starchy/animal-origin dietary pattern was associated with adverse adiposity and blood pressure indicators, while a plant-based pattern was associated with more favorable adiposity and blood pressure profiles. This indicates potential target areas for preventing metabolic syndrome through effective dietary intervention in this population. More large-scale prospective studies are needed to further examine the relationship between dietary patterns and metabolic syndrome among youth in Sudan.

## Data Availability

The original contributions presented in the study are included in the article/supplementary material, further inquiries can be directed to the corresponding author/s.
